# SnipViz: a compact and lightweight web site widget for display and dissemination of multiple versions of gene and protein sequences

**DOI:** 10.1186/1756-0500-7-468

**Published:** 2014-07-23

**Authors:** Daniel Jaschob, Trisha N Davis, Michael Riffle

**Affiliations:** 1 Department of Biochemistry, University of Washington, Seattle, UW Box 357350, 1705 NE Pacific St., Seattle, WA 98195-7350, USA; 2 Department of Genome Sciences, University of Washington, Seattle, UW Box 357350, 1705 NE Pacific St., Seattle, WA 98195-7350, USA

## Abstract

**Background:**

As high throughput sequencing continues to grow more commonplace, the need to disseminate the resulting data via web applications continues to grow. Particularly, there is a need to disseminate multiple versions of related gene and protein sequences simultaneously—whether they represent alleles present in a single species, variations of the same gene among different strains, or homologs among separate species. Often this is accomplished by displaying all versions of the sequence at once in a manner that is not intuitive or space-efficient and does not facilitate human understanding of the data. Web-based applications needing to disseminate multiple versions of sequences would benefit from a drop-in module designed to effectively disseminate these data.

**Findings:**

SnipViz is a client-side software tool designed to disseminate multiple versions of related gene and protein sequences on web sites. SnipViz has a space-efficient, interactive, and dynamic interface for navigating, analyzing and visualizing sequence data. It is written using standard World Wide Web technologies (HTML, Javascript, and CSS) and is compatible with most web browsers. SnipViz is designed as a modular client-side web component and may be incorporated into virtually any web site and be implemented without any programming.

**Conclusions:**

SnipViz is a drop-in client-side module for web sites designed to efficiently visualize and disseminate gene and protein sequences. SnipViz is open source and is freely available at https://github.com/yeastrc/snipviz.

## Background

Web sites designed to disseminate data that annotate gene or proteins frequently also disseminate sequences for the respective genes or proteins. Where there is only a single sequence, the problem of dissemination is relatively simple. The sequence is displayed as plain text in its entirety using a fixed-width font and may optionally be formatted so that position number in the sequence can be easily determined. For example, the sequence for the protein DSN1 from *S. cerevisiae* may be displayed as the following:

However, there is often a need to simultaneously disseminate multiple versions of related sequences. Examples include displaying sequences for a given protein from many strains of a particular species of yeast, displaying multiple alleles from the same gene across a population, and displaying the results of a multiple sequence alignment of a homologous protein across many species. While the above format works well for displaying a single sequence, it is not well suited for simultaneously displaying multiple sequences in a way that is easily read by humans.

Strategies for simultaneously displaying multiple sequences on the web include displaying FASTA-formatted data [1],where each version of the sequence is sequentially displayed in its entirety as plain text; or more commonly, displaying ClustalW-formatted data [2], where the first N positions of each aligned sequence is listed sequentially in some order, then the next N positions, then the next, until all versions of the entire aligned sequences have be displayed. On web pages, it is common to color code ClustalW-format data based on sequence variability at specific positions or based on some property of nucleotides or amino acids. However, this method rapidly becomes cumbersome, space-inefficient, and incomprehensible to human readers as more versions of the sequence are included and as the sequence gets longer.

Many programs exist for generating or viewing multiple sequence alignment information [3-8]. Miew [6] is of particular note as very mature and feature-rich program designed specifically for the display of multiple sequence information. Mview supports several input formats, is highly configurable with regard to output, and supports saving the output as HTML that may be used to display the data in a web page. However, this HTML is a static display of the data that is subject to the same limitations described above. Additionally, Mview requires running an external program, either in advance or at runtime, which adds to the complexity. The JalviewLite applet [7] is a feature-rich Java applet that may be used by websites to disseminate multiple sequences using a dynamic interface. In this interface, the sequences may be presented using a sliding window, which eliminates the space and legibility problems created by statically displaying the entire sequence. However, JalviewLite requires that the end user have Java and Java web browser plugins installed. As a general-purpose dissemination platform, this is less than ideal as not every user running a web browser has Java installed and configured and Java applets are not compatible with many portable devices. Ideally, the data would be presented using a dynamic interface that runs entirely within the web browser and requires no external plugins.

Here we present SnipViz, designed to efficiently disseminate many versions of sequences of any length on the web. SnipViz makes use of standard dynamic HTML and JavaScript to present an interactive sliding window view of the aligned sequences, so that increasing sequence sizes do not result in more space on the web page being devoted to displaying the sequences. SnipViz supports hierarchical clustering of sequences, color coding of positions, and graphical whole-sequence display to assist in finding and interpreting locations of sequence variation. SnipViz may be integrated into a web page using standard HTML without any programming. SnipViz is open source and freely available at https://github.com/yeastrc/snipviz.

## Findings

### Implementation

#### Web component

SnipViz is implemented using standard World Wide Web technologies: JavaScript, AJAX, HTML, and Cascading Style Sheets (CSS). It is cross platform and has been tested in current versions of Chrome, Firefox, Safari, and Internet Explorer running on Windows, Linux, MacOS, and iOS. SnipViz has no server-side component--other than the availability over standard HTTP of the data to be displayed.

SnipViz makes use of 3^rd^ party JavaScript libraries, including jQuery versions 1.5.2 and up (http://jquery.com/), jQuery UI versions 1.7.0 and up (http://jqueryui.com/), the JavaScript vector graphics library (http://www.walterzorn.de/en/jsgraphics/jsgraphics_e.htm) and DHTML tooltips library (http://www.walterzorn.de/en/tooltip/tooltip_e.htm) by Walter Zorn.

#### Architecture

The web page initializes SnipViz by indicating the location of the input data using standard HTML. JavaScript code is then executed on the end-user client that retrieves the indicated data via HTTP, parses the data, then constructs and displays the graphical user interface (GUI) on the web page. Once loaded, the GUI is purely a client-side application and no further communication with the server takes place.

#### Input formats

The only required data for SnipViz are either DNA or protein sequence data in FASTA format. SnipViz must be configured with the location of the FASTA data (either a static file or the output of a dynamic program) that contains all of the sequences and their associated labels. If the sequences require alignment, the sequences must be aligned prior to being loaded.

SnipViz may optionally display a dendrogram indicating the hierarchical clustering of the sequences based on any property the implementer wishes (e.g., phylogeny of originating organisms or similarity of sequences being displayed). To display the dendrogram, the user must indicate the location of Newick-formatted data [9] (either static file or output of a dynamic program) that contains the desired clustering for all of the labels in the FASTA sequence file.

### Installation

SnipViz is incorporated into a web page by importing JavaScript files and specifying data locations using standard HTML. After importing the necessary JavaScript, the following is an example of the HTML necessary to configure and run SnipViz on a web site.

This HTML will result in an instance of SnipViz appearing wherever this HTML is placed on the web page, displaying the indicated data—in this case, hierarchically clustered DNA sequences from the indicated Newick and FASTA files.

SnipViz may be optionally configured with both DNA and protein sequences at the same time and will automatically provide a link for toggling between viewing the DNA and protein sequences. This is accomplished by indicating the locations of the DNA and protein sequences in the same configuration block. For example:

### Graphical user interface

#### Basic functionality

**Figure 1:**
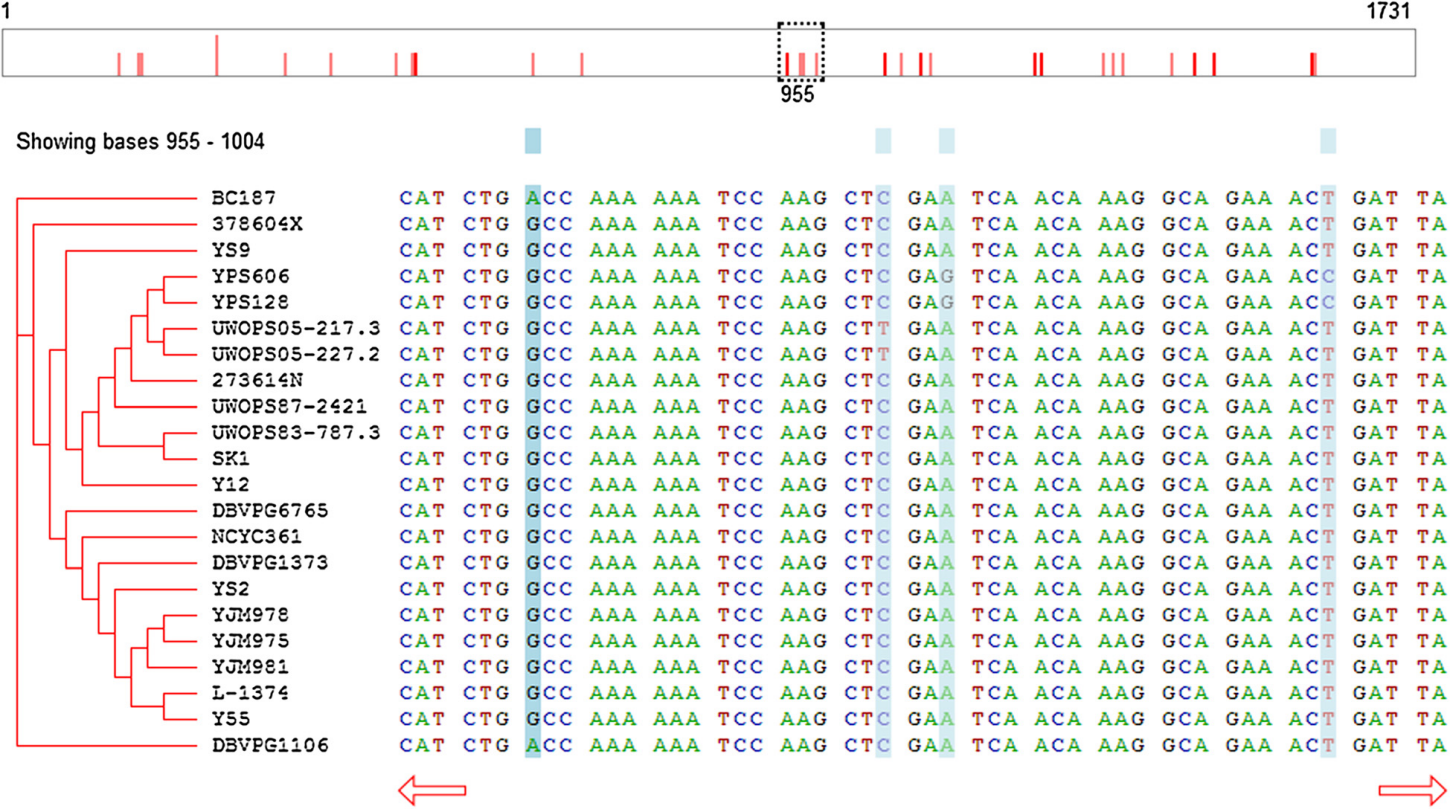
**A screen capture of SnipViz displaying the DNA sequence of a gene for 22 different strains of*S. cerevisiae*.** The top rectangle is a graphical representation of the whole sequence (1,731 nucleotides long) that serves as a whole-sequence navigation bar. The dashed box indicates the currently-viewed segment of the sequence and may be clicked and dragged to the desired location in the sequence. The red bars indicate locations of variation in the sequence among all the strains. The bottom left displays a hierarchically clustered list of the sequence labels, and the bottom right displays the sequences. The blue bars highlight columns in the sequence display where variation occurs.

A screenshot of the SnipViz GUI is shown in Figure 1. At the top of the interface is a graphical representation of the whole sequence with red bars indicating locations of sequence variation. In this representation is a dashed box that represents the part of the sequence currently being displayed below. Users may click anywhere in this sequence representation to center the currently-viewed window on that location, or click and drag the dashed box to move the window.

Beneath the whole sequence representation is the display of the sequences, themselves. To the left are the labels supplied for the sequences from the FASTA file and, optionally, the dendrogram representing the hierarchical clustering of the sequences from a Newick file. To the right the section of the sequences corresponding to the currently-viewed window are displayed. Beneath the sequences, users may click the arrows to page left or right through the sequences.

#### Sequence highlighting

**Figure 2:**
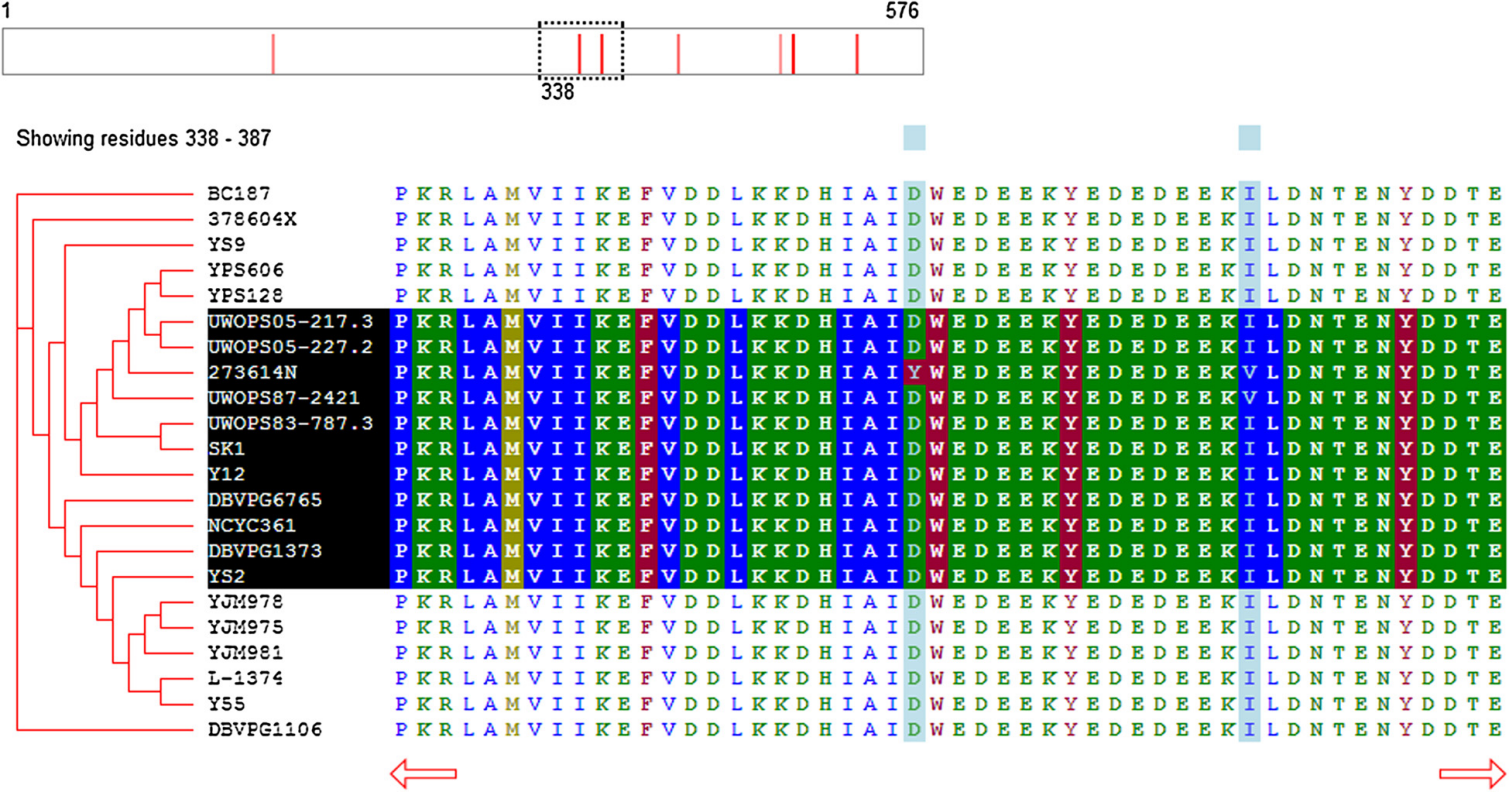
**A screen capture of SnipViz illustrating the effect of highlighting specific sequences.** In this example, the sequence for a protein from 22 different strains of *S. cerevisiae* is shown. The user has clicked the names of highlighted sequences to enable highlighting of those sequences. The red lines in the sequence navigation bar and blue column highlights of the sequence now indicate locations of sequence variability only among the highlighted sequences.

Users may click the labels to toggle highlighting of one or more sequences. (Figure 2) If highlighted, a sequence will have its colors inverted to emphasize the sequence and make sequence variation among highlighted sequences easier to discern. If more than one sequence is highlighted, the indicators of sequence variation (in the whole-sequence representation above the sequences and the column highlighting in the sequence window) will only indicate variation among the highlighted sequences.

#### Very long sequences

**Figure 3:**
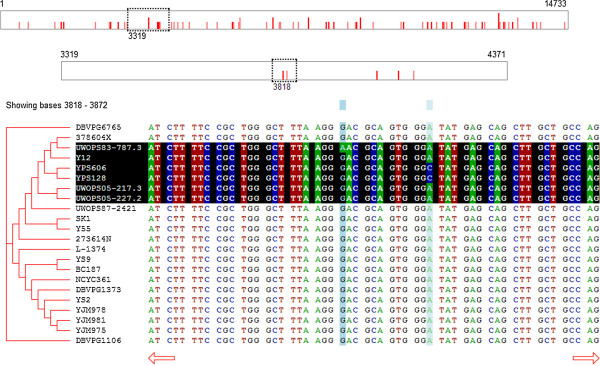
**A screen capture of SnipViz illustrating the effect of viewing very long sequences; in this case 22 separate DNA sequences each 14,733 nucleotides in length.** A second sequence navigation bar has been created at the top, such that the dashed box in the top bar indicates the region of the sequence shown in the second bar, and the dashed box in the second bar indicates the region of the sequence being displayed below. Both boxes may be clicked and dragged in their respective sequence navigation bars to display the DNA sequence at the desired location.

Because the dashed box in the whole-sequence window represents the currently-viewed segment of the sequence, and because that segment is a fixed size (e.g., 50 nucleotides), the width of the dash box will change based on the length of the whole sequence. As the overall sequence gets longer, the dashed box representing the segment of the sequence being shown will become narrower. If the sequence is long enough, the box representing 50 positions in that sequence will be so narrow that it will not be a useful element of the GUI.To solve this problem, SnipViz will detect when the dashed box would be too narrow and employ a second level graphical sequence representation (Figure 3). When this occurs, the top sequence representation will contain a dashed box that indicates which segment of the sequence is represented by the sequence representation below it. This second sequence representation will itself contain a dashed box that indicates which segment of the sequence is being displayed in the sequence viewer area below. This method ensures that even extremely long sequences may be graphically represented and simply navigated.

#### Indicators of variation

As previously mentioned, the whole-sequence representation above the sequences contains red lines that serve as indicators of locations of sequence variation among all of the sequences or among the currently-highlighted sequences. Both the shade of red and the height of the line contain information.

The height of the line indicates the number of positions represented by that line that contain variation. Because the width of the graphical whole-sequence representation may contain fewer pixels than the number of positions in the sequence, each line may represent more than one position in the sequence. A taller line indicates more relative variation at the represented position in the sequence than a shorter line.

The shade of red is meant to indicate the significance of the variation at a given position, with darker red indicating more significant variation. In the case of DNA sequences, there are two shades of red: pale and dark. Pale red indicates that all variation in that position result in the same amino acid being encoded (silent mutations). Dark red indicates that there is at least one substitution at that position among all the sequences that results in a different encoded amino acid. In the case of protein sequences, the shade of red is determined by a calculation using the BLOSUM 80 [10] amino acid substitution matrix. For all positions with sequence variation, a score is calculated by comparing all amino acids at that position against all other amino acids at that position and summing the BLOSUM 80 substitution values. The resulting values are used to linearly scale the intensity of red in the indicator line between a pale and dark shade of red.

The main sequence viewing area also contains indicators of location of sequence variability. These appear as a shade of blue that highlights specific columns in the sequence and appears as an indicator block above the column. The shade of blue is determined using the same logic as the shade of red in the indicator lines described above.

### Implementation considerations

Although there is no limit coded into Snipviz for the length or number of sequences that may be simultaneously viewed, there are practical limitations that should be considered. Increasing the length or number of sequences consumes resources and places an increasingly large computational load on Javascript and the web browser when the user manipulates the interface. To determine practical limitations, the responsiveness of Snipviz was tested using latest versions of Chrome, Firefox, and Internet Explorer on Microsoft Windows 7 by varying the length (up to 120,000 positions) and number of displayed sequences (up to 1,000). For all three browsers we found that displaying up to 100 sequences resulted in acceptable performance (the length of the sequences had a negligible impact). Snipviz successfully loaded in all of our tests (up to 1,000 sequences each with 120,000 positions), though responsiveness of the interface was severely degraded by this point. Loading thousands of sequences is not recommended, as limitations of available memory start to become a significant issue that may result in crashing of the web browser.

### Current implementations

To view simple demonstrations of implementations of SnipViz, see http://www.yeastrc.org/snipviz/. There are demonstrations of multiple configurations (DNA, protein, very long sequences, and clustering), and each demonstration includes the HTML required to implement it.

To see an example of how SnipViz may be integrated into a web application, see http://www.yeastrc.org/g2p/, a web application developed to support a study examining the phenotypic consequences of sequence variation among 22 phylogenetically diverse strains of the budding yeast *S. cerevisiae*[11]*.* For this implementation, SnipViz is used to visualization locations of sequence variation in the same gene or protein across all of the sequences strains of yeast. For a specific example, see http://www.yeastrc.org/g2p/phenomeviewProtein.do?orfName=YCR088W&listing=ABP1+%2f+YCR088W.

### Future directions

Central among the future plans for SnipViz are (1) removing the requirement that the loaded sequences be either DNA or protein sequences and (2) making the highlighting system more modular so that custom logic may be easily used to override the default highlighting system. Not only should users be able to display RNA or, indeed, any conceivable type of sequence, they should be able to easily implement some highlighting that indicates their own determination of significance for variability at specific positions.

We welcome other developers to download the code, make improvements, and contribute to the project at https://github.com/yeastrc/snipviz.

## Conclusions

Snipviz is a client-side web module for efficiently displaying multiple versions of DNA or protein sequences. It has a highly dynamic, interactive graphical user interface written using standard World Wide Web technologies and may be simply installed without any programming being necessary. It is cross-platform and compatible with all current web browsers. SnipViz is open source and freely available at https://github.com/yeastrc/snipviz.

## Availability and requirements

**Project name:** SnipViz

**Project home page:**https://github.com/yeastrc/snipviz

**Operating system(s):** Platform independent

**Programming language:** JavaScript, HTML, CSS

**Other requirements:** None

**License:** Apache 2.0

**Any restrictions to use by non-academics:** None

## Abbreviations

AJAX: Asynchronous JavaScript and XML; CSS: Cascading Style Sheets; GUI: Graphical User Interface; HTTP: HyperText Transfer Protocol; HTML: HyperText Markup Language; XML: Extensible Markup Language.

## Competing interests

The authors declare that they have no competing interests.

## Authors’ contributions

DJ performed all programming, set up the source code repository, and prepared user documentation. TND provided scientific input, feedback, and support. MR conceived of the software, designed the GUI, directed development, and prepared the manuscript. All authors read and approved the final manuscript.
